# Post‐Thrombotic Syndrome of Lower Extremity

**DOI:** 10.1002/ccr3.73152

**Published:** 2026-07-14

**Authors:** Dou dou Li, Wen Chen, Ba‐yi Liu, Hua‐min Wang, Bao‐ping Xu

**Affiliations:** ^1^ Department of Critical Care Medicine Zhongshan Hospital of Traditional Chinese Medicine Affiliated to Guangzhou University of Traditional Chinese Medicine Guangzhou Zhongshan China; ^2^ Department of Orthopedics, Longhua Hospital Shanghai University of Traditional Chinese Medicine Shanghai China

**Keywords:** anticoagulation therapy, deep vein thrombosis, in‐stent restenosis, post‐thrombotic syndrome, venous stenting

## Abstract

Post‐thrombotic syndrome (PTS) is a chronic complication of deep vein thrombosis (DVT) that imposes significant morbidity, reduces quality of life, and is costly. We, herein, present a case report of a patient who developed PTS following DVT in the lower extremities. The clinical characteristics observed in this patient are highly representative and hold substantial clinical significance.

**Key Clinical Message**

Post‐thrombotic syndrome (PTS) is the most common chronic complication following deep vein thrombosis (DVT). PTS can lead to long‐term pain, varicose veins, reduced work capacity, and increased healthcare burden. Early standardized management is crucial, and novel oral anticoagulants may help reduce the incidence of PTS.

A 68‐year‐old female patient who presented with a longstanding history of swelling in the left lower extremity, persisting for over 9 years, was admitted to our hospital due to a five‐day history of worsened symptoms. Following a cerebral hemorrhage 9 years prior, the patient developed persistent swelling and unrelieved pain in the left lower extremity. A color Doppler ultrasonography conducted at an external facility identified the presence of iliac vein thrombosis in the left lower extremity. Consequently, an inferior vena cava (IVC) filter was inserted to mitigate the risk of pulmonary embolism. After stabilization of the cerebral hemorrhage, the patient was discharged with a prescription for oral rivaroxaban at a dosage of 20 mg once daily for anticoagulation therapy. Six months subsequently, the rivaroxaban dosage was reduced to 10 mg once daily. Two years prior, the patient experienced exacerbated swelling in the left lower extremity. Computed tomography (CT) angiography performed at another institution diagnosed an occlusion of the left iliac vein, leading to the placement of a stent in the affected vein. Postoperatively, the patient was prescribed oral rivaroxaban at 10 mg once daily for anticoagulation; however, adherence to the anticoagulant regimen was inconsistent. 1 year ago, follow‐up CT venography of the lower extremity revealed an occlusion of the left iliac vein stent, for which no specific treatment was administered. The patient's lower extremity edema progressively deteriorated, characterized by pronounced thickening of the left lower limb. Five days prior, the patient experienced a significant exacerbation of the swelling in the left lower limb, accompanied by severe pain, and was subsequently brought to the emergency department of our hospital by family members. The patient reported no personal history of other diseases, no family history of venous thrombosis, and no history of congenital vascular malformations.

On her admission physical examination, swelling was observed in the left waist and buttock regions and a significant number of varicose veins were observed on the left abdominal and pelvic walls. The left lower extremity demonstrated severe edema with significant hyperpigmentation, local slight rise of temperature, and tenderness. The pulsations of the dorsalis pedis and posterior tibial arteries in the left lower extremity were significantly diminished and nearly imperceptible. The circulation function in the lower extremity was impaired, with localized skin ulceration evident (Figure 1). The patient's Villalta score was 25, indicating severe post‐thrombotic syndrome. X‐ray angiography of the left lower extremity was performed, demonstrating insufficient visualization of the deep veins, but numerous collateral circulations were noted (as shown in Figure 2). After admission, symptomatic treatments including anti‐infective therapy, fluid replacement, blood transfusions, and supplementing protein were conducted. The patient felt that the pain of the left lower extremity was significantly relieved, but the patient's left whole lower extremity shows diffuse swelling with hyperpigmentation. She was discharged on the tenth day with continuation of rivaroxaban 20 mg daily. During a follow‐up 1 week post‐discharge, the patient continued to exhibit swelling in the lower extremities, although no significant pain was reported.

**FIGURE 1:**
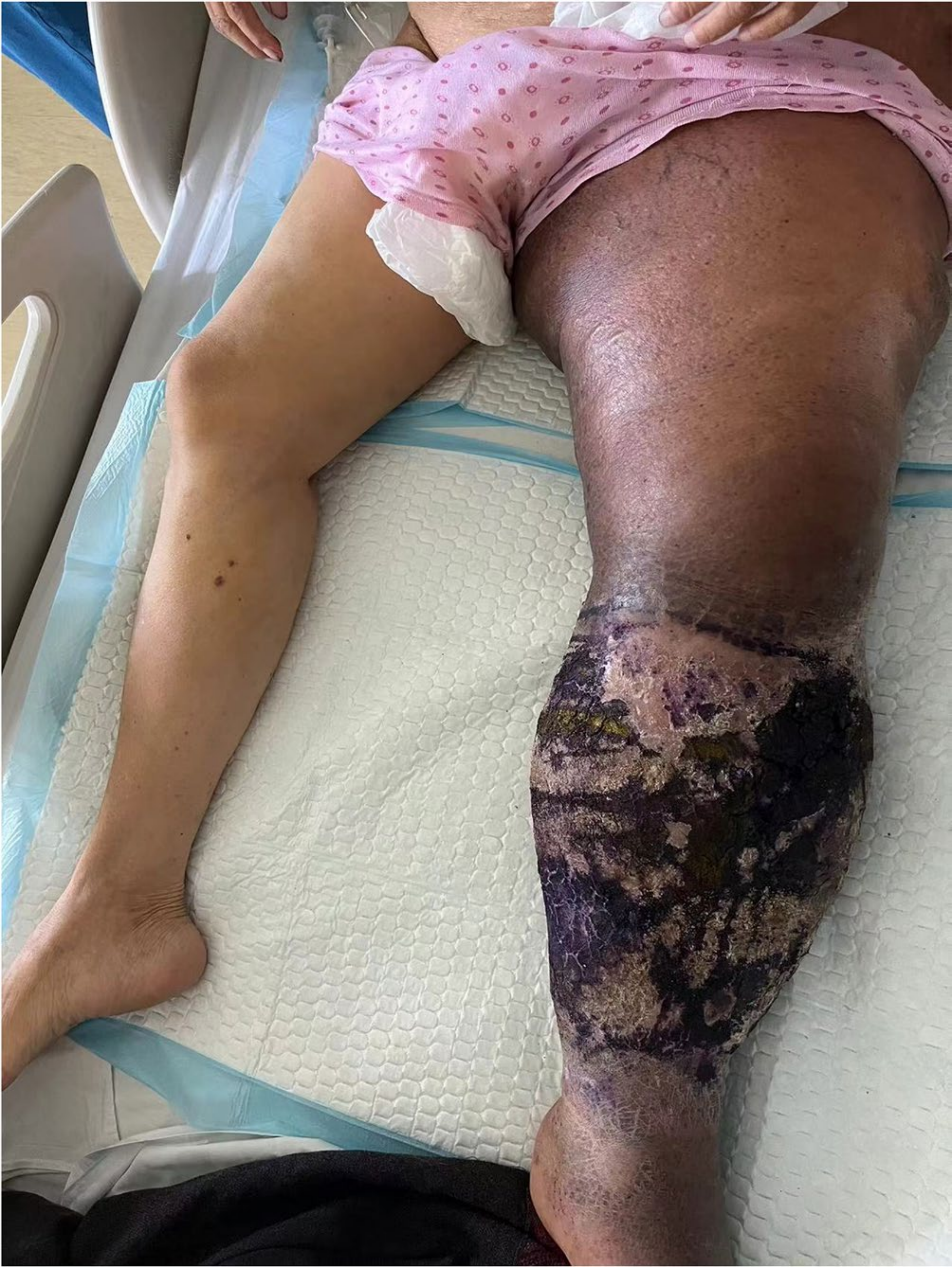
A comparison of both lower limbs showing hyperpigmentation and severe edema in the left lower limb along with local skin ulceration while the right lower limb is normal.

**FIGURE 2:**
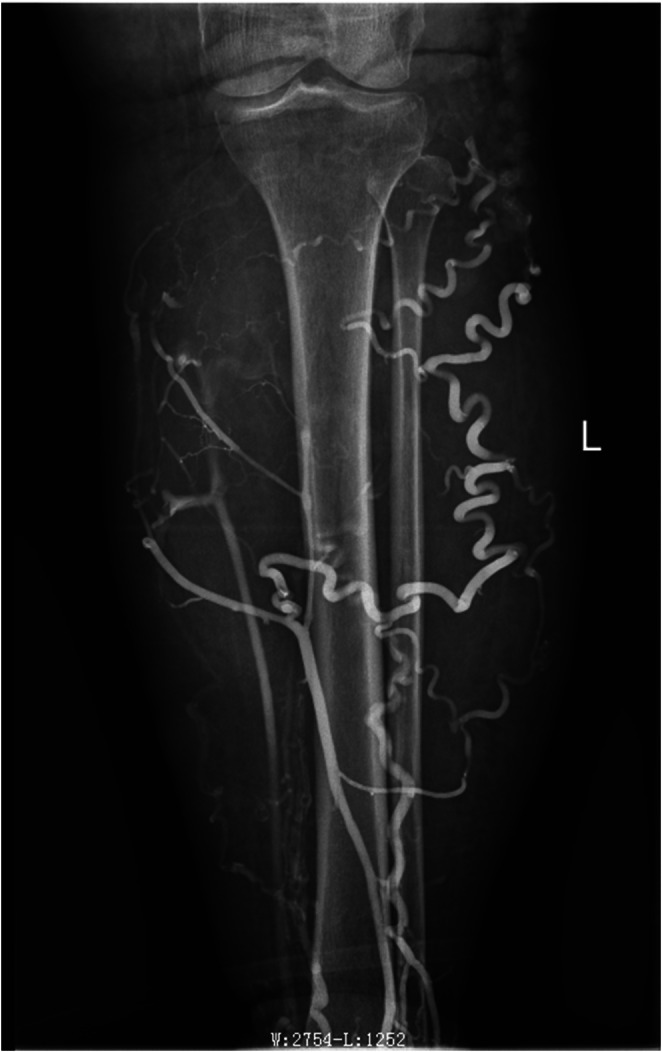
A X‐ray angiography of the left lower extremity indicated that the left anterior tibial vein, posterior tibial vein, and popliteal vein were not clearly visualized. Additionally, the formation of multiple collateral circulations was observed in the left lower extremity.

Post‐thrombotic syndrome (PTS) is a chronic complication of deep venous thrombosis (DVT), characterized by venous obstruction, resulting from the thrombus occluding the venous vessels, and venous reflux, due to the compromised function of the deep venous valves. This condition manifests as a constellation of symptoms, including edema, pain, skin hyperpigmentation, and recalcitrant skin ulcers, which arise from chronic venous hypertension and impaired venous return in the affected limb. Symptoms of PTS usually occur within 3–6 months after DVT, but about 50% of individuals can occur up to 2 years after DVT. The prevalence of PTS in the population is expected to increase as the number of adults with venous thromboembolism continues to grow [1]. Despite adherence to standard anticoagulant therapy, the incidence of PTS remains substantial, ranging from 30% to 50%. Furthermore, severe manifestations of PTS are observed in 5% to 10% of patients with DVT [1].

The use of venous stents offers a new therapeutic possibility for PTS, demonstrating favorable effects in improving venous patency and alleviating symptoms, thereby significantly enhancing patients' quality of life [2]. Although endovenous stenting has emerged as the method of choice to treat lower‐extremity venous outflow obstruction and is used in patients with established PTS after previous DVT to reduce symptoms of chronic pain and swelling and to aid ulcer healing in severe cases [3]. However, it must be recognized that venous stenting is effective only in a subset of PTS patients. In‐stent restenosis (ISR) remains a common and challenging complication following venous stenting for PTS and is associated with multiple factors, including adherence to post‐procedural anticoagulation therapy, thrombus burden, and impaired blood flow; the treatment of PTS in the lower extremities is still limited. Furthermore, studies have indicated that total stent length is not a decisive factor influencing patency rates, suggesting that an individualized treatment strategy based on specific lesion characteristics should be adopted when placing stents. In summary, venous stenting has significant clinical value in the management of PTS. In clinical practice, strict patient selection is essential, and individualized treatment plans should be developed based on each patient's specific condition, along with enhanced post‐procedural management to reduce the risk of re‐intervention.

## Author Contributions

**Dou dou Li:** conceptualization, writing – original draft. **Wen Chen:** conceptualization, writing – original draft. **Hua‐min Wang:** data curation. **Ba‐yi Liu:** data curation. **Bao‐ping Xu:** conceptualization, writing – review and editing.

## Funding

The authors have nothing to report.

## Consent

Written informed consent for publication of their clinical details and/or clinical images was obtained from the patient.

## Conflicts of Interest

The authors declare no conflicts of interest.

## Data Availability

The data were available upon appropriate requests from the corresponding author.
